# A Novel Time Lag Method for the Analysis of Mixed Gas Diffusion in Polymeric Membranes by On-Line Mass Spectrometry: Pressure Dependence of Transport Parameters

**DOI:** 10.3390/membranes8030073

**Published:** 2018-09-03

**Authors:** Marcello Monteleone, Elisa Esposito, Alessio Fuoco, Marek Lanč, Kryštof Pilnáček, Karel Friess, Caterina Grazia Bezzu, Mariolino Carta, Neil Bruce McKeown, Johannes Carolus Jansen

**Affiliations:** 1 Institute on Membrane Technology (ITM-CNR), Via P. Bucci 17/C, 87036 Rende (CS), Italy; m.monteleone@itm.cnr.it (M.M.); e.esposito@itm.cnr.it (E.E.); a.fuoco@itm.cnr.it (A.F.); 2 Department of Physical Chemistry, University of Chemistry and Technology, Technická 5, 166 28 Prague, Czech Republic; marek.lanc@vscht.cz (M.L.); krysot@gmail.com (K.P.); karel.friess@vscht.cz (K.F.); 3 EastChem, School of Chemistry, University of Edinburgh, David Brewster Road, EH9 3FJ Edinburgh, UK; BezzuC@cardiff.ac.uk (C.G.B.); Neil.McKeown@ed.ac.uk (N.B.M.); 4 Department of Chemistry, College of Science, Swansea University, Grove Building, Singleton Park, SA2 8PP Swansea, UK; mariolino.carta@swansea.ac.uk

**Keywords:** Time lag, diffusion coefficient, mixed gas permeation, membrane, gas mixture, on-line mass spectrometry, polymer of intrinsic microporosity, PIM

## Abstract

This paper presents a novel method for transient and steady state mixed gas permeation measurements, using a quadrupole residual gas analyser for the on-line determination of the permeate composition. The on-line analysis provides sufficiently quick response times to follow even fast transient phenomena, enabling the unique determination of the diffusion coefficient of the individual gases in a gas mixture. Following earlier work, the method is further optimised for higher gas pressures, using a thin film composite and a thick dense styrene-butadiene-styrene (SBS) block copolymer membrane. Finally, the method is used to calculate the CO_2_/CH_4_ mixed gas diffusion coefficients of the spirobisfluorene-based polymer of intrinsic microporosity, PIM-SBF-1. It is shown that the modest pressure dependence of the PIM-SBF-1 permeability can be ascribed to a much stronger pressure dependence of the diffusion coefficient, which partially compensates the decreasing solubility of CO_2_ with increasing pressure, typical for the strong sorption behaviour in PIMs. The characteristics of the instrument are discussed and suggestions are given for even more versatile measurements under stepwise increasing pressure conditions. This is the first report on mixed gas diffusion coefficients at different pressures in a polymer of intrinsic microporosity.

## 1. Introduction

Gas separation by polymeric membranes is a well-established industrial process for a number of applications and at the same time it is a vivid research field [1]. Significant effort is dedicated to the search for novel membrane materials with improved performance with respect to state-of-the-art commercial membranes. For successful development of novel membrane materials, the detailed knowledge of their transport properties is required. In this light, sorption, permeation, and modelling studies are all used to determine the basic transport parameters: the permeability, solubility, and diffusion coefficient. Most of those studies are dedicated to the transport properties of pure gases in novel materials, and much less work is focused on gas or vapour mixtures via experimental permeation [2,3,4] and sorption [5,6,7] measurements or via modelling [8,9,10] of these processes. Whereas the measurement of permeability is a routine technique, only very few reports are available on the diffusion of gas mixtures because this requires more sophisticated methods to study the transient phase in the permeation process. Proposed methods based on NMR spectroscopy to study diffusion are very powerful [11] and can be applied to mixtures [12], but are not easily integrated in a permeation process. Selective condensation of one of the components of a gas mixture during the permeation transient [13] provides valuable insight into the kinetics, but is not suitable for routine analysis of membranes. Instead, on-line mass spectrometry is versatile and has been successfully used to follow transient phenomena during pervaporation [14,15,16,17] and gas permeation experiments [18,19,20] of relatively slow membranes. We used a similar setup for permeation measurements of polymers of intrinsic microporosity, but since they have very high permeability and very fast kinetics, we have only obtained their steady-state permeation data with this method so far [21,22,23,24].

Of all methods to determine the transport parameters of nonporous polymeric membranes, the time lag method introduced by Daynes a century ago [25] remains by far the most popular method because of its experimental simplicity and because of the easy interpretation of the results. The diffusion coefficient is most commonly measured with a so-called fixed-volume/pressure increase instrument, measuring the time lag, *Θ*, from the permeation transient and steady state [26]:

(1) $$\Theta =\frac{l^{2}}{6D_{i}}$$

where *l* is the membrane thickness (m) and *D* is the diffusion coefficient (m^2^ s^−1^). A practical limitation is that for a correct application of this method, the measurements should obey a number of very strict criteria. One of the most important conditions is that the gas solubility and the diffusion coefficient should be constant over the experimental pressure range [27]. The simplest linear model further requires the total absence of the permeant in the membrane at the beginning of the experiment and negligible concentration of the permeant in the permeate side during the experiment. The latter is fundamentally impossible because a finite pressure increase is needed to measure the permeability, and this may lead to errors if the pressure increase is not kept as low as possible [27,28]. Changing boundary conditions at the feed and/or the permeate side requires a significantly more complex combined experimental and numerical approach [29,30]. Some example solutions for situations with changing boundary conditions are given in the review of Rutherford et al. [31]. Alternatively, fully computational methods may be used [32]. Most membrane materials do not have linear sorption behaviour and Al-Qasas et al. recntly proposed a method for the characterization of membranes with strong dual mode behaviour, taking also into account the changing boundary conditions in the feed and permeate side [29]. They quantified how dual mode sorption parameters affect the correctness of time lag measurements and, although the effect of nonlinear sorption is significant, they concluded that this does not exclude accurate analysis of the transport parameters by the classical time lag method [28]. Thus, the effective transport parameters are obtained, averaged over the thickness of the membrane.

For the fixed volume time-lag setup for single gases in the present work, the entire time lag curve and the permeation curve in steady state are given by the equations [33]:

(2) $$p_{t}=p_{0}+{\left(\frac{dp}{dt}\right)}_{0}\cdot t+\frac{RT\cdot A\cdot l}{V_{P}\cdot V_{m}}\cdot p_{f}\cdot S\cdot \left(\frac{D\cdot t}{l^{2}}-\frac{1}{6}-\frac{2}{\pi^{2}}\sum_{1}^{\infty }\frac{{\left(-1\right)}^{n}}{n^{2}}\exp\left(-\frac{D\cdot n^{2}\cdot \pi^{2}\cdot t}{l^{2}}\right)\right)$$

(3) $$p_{t}=p_{0}+{\left(\frac{dp}{dt}\right)}_{0}\cdot t+\frac{RT\cdot A}{V_{P}\cdot V_{m}}\cdot \frac{p_{f}\cdot S\cdot D}{l}\left(t-\frac{l^{2}}{6D}\right)$$

in which *p_t_* is the permeate pressure (bar) at time, *t* (s), *R* is the universal gas constant (8.314 × 10^−5^ m^3^ bar mol^−1^·K^−1^), *T* is the absolute temperature (K), *A* is the exposed membrane area (m^2^), *V_P_* is the permeate volume (m^3^), *V_m_* is the molar volume of a gas at standard temperature and pressure (22.41 × 10^−3^ m^3^_STP_ mol^−1^ at 0 °C and 1 atm), *p_f_* is the feed pressure (bar), *S* is the gas solubility (m^3^_STP_ m^−3^ bar^−1^), and *D* is the diffusion coefficient (m^2^ s^−1^). In the presence of minor leaks, *p*_0_ would be the starting pressure (bar) and (*dp/dt*)_0_ would be the baseline slope (bar s^−1^), but normally these terms are negligible.

For a constant pressure/variable volume system, Ziegler et al. [34] suggested an equation expressing the change in the permeate flow rate upon a step change in the feed concentration. In the present case, where we use the cumulative volume of permeating gas, Equations (2) and (3) can be easily converted to express the total permeate volume, *V*_t,STP_, in time:

(4) $$V_{t,STP}=V_{0}+{\left(\frac{dV}{dt}\right)}_{0}\cdot t+A\cdot l\cdot p_{f}\cdot S\cdot \left(\frac{D\cdot t}{l^{2}}-\frac{1}{6}-\frac{2}{\pi^{2}}\sum_{1}^{\infty }\frac{{\left(-1\right)}^{n}}{n^{2}}\exp\left(-\frac{D\cdot n^{2}\cdot \pi^{2}\cdot t}{l^{2}}\right)\right)$$

(5) $$V_{t,STP}=V_{0}+{\left(\frac{dV}{dt}\right)}_{0}\cdot t+\frac{A\cdot p_{f}\cdot S\cdot D}{l}\cdot \left(t-\frac{l^{2}}{6D}\right)$$

where the membrane time lag is then given by the intercept between the extrapolated baseline curve (*V*_0_ + *t*·(*dV/dt*)_0_, which should be zero in a leak free system with a defect-free membrane), and that of the cumulative permeate volume versus time at steady state.

Recently, we have optimised our constant pressure/variable volume system and the measurement procedures, based on a mass spectrometric residual gas analyser for the on-line analysis of the permeate gas, even during the transient phase in thin films or highly permeable membranes [35]. Careful identification of all instrumental parameters and extensive error analysis allowed the accurate calculation of the mixed gas diffusion coefficient in Rubbery Pebax^®^ membranes, glassy Hyflon^®^ perfluoropolymer membranes, and the polymer of intrinsic microporosity, PIM-EA-TB. For a system with on-line measurement of the permeate composition, the determination of the time lags of the individual components in the mixture, *Θ_i_*, must take into account the delay in the response due to the dead volume in the system:

(6) $$\Theta_{i}=\Theta_{0}+\frac{l^{2}}{6D_{i}}$$

where *Θ_0_* is the instrumental time lag, related to the tube, cell, and analyser volumes in the instrument, as well as the response of the electronics; and *D_i_* is the diffusion coefficient of the individual gas species, *i*. As discussed previously, the instrumental time lag can be expressed by the following equation [35]:

(7) $$\Theta_{0}=\frac{V_{Feed}}{\phi_{Feed}}+\frac{V_{Downstream}}{\phi_{Downstream}}+\frac{V_{Inlet}}{\phi_{Inlet}}$$

where *V_Feed_*, *V_Downstream_*, and *V_Inlet_* are the volume of the feed side, the volume of the permeate side until the sampling point, and the volume of the injection line, respectively. The terms *φ_Feed_*, *φ_Downstream_*, and *φ_Inlet_* indicate the respective total volumetric flow rates in those sections of the setup. In the case of very low permeate fluxes, *φ_Downstream_*, is practically equal to the sweep flow rate. Thus, for the total time lag:

(8) $$\Theta_{i}=\Theta_{0}+\Theta_{Mem,i}=\frac{V_{Feed}}{\phi_{Feed}}+\frac{V_{Downstream}}{\phi_{Downstream}}+\frac{V_{Inlet}}{\phi_{Inlet}}+\frac{l^{2}}{6D_{i}}$$

where *Θ_Mem,i_* represents the time lag of the membrane, equal to *l*^2^/6*D*. At atmospheric feed and permeate pressure, and with 200 cm^3^ min^−1^ feed and 30 cm^3^ min^−1^ sweep flow rates, the total instrumental time lag was found to be approximately 20 s, and this value must be subtracted from the total time lag to determine the time lag related to the membrane transport, and thus to calculate the effective gas diffusion coefficient [35].

**Figure 1:**
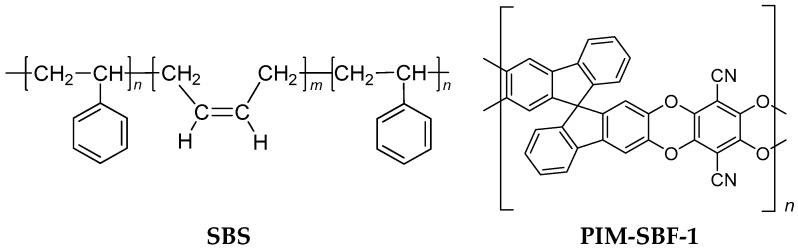
Structural formulas of Styrene-Butadiene-Styrene triblock copolymer (SBS) and the polymer of intrinsic microporosity, PIM-SBF-1 [36,37].

The scope of the present manuscript is to further improve the method, enabling transient and steady state permeation measurements for subsequent calculation of the mixed gas diffusion coefficients also at higher pressures, without compromising the accuracy of the method. Two different approaches will be presented: The first is based on individual measurements with instantaneous exposure of the membrane to the gas mixture at different pressures; the second is based on measurements with a step-wise increase in the feed pressure. The critical factors in the permeation setup and in the experimental procedures will be carefully investigated with a poly(styrene-*b*-butadiene-*b*-styrene) block copolymer to allow optimization of the operational parameters. The method is then applied to the polymer of intrinsic microporosity, PIM-SBF-1 (Figure 1), with the CO_2_/CH_4_ mixture as a demonstration of the suitability of this method, even in the case of strongly pressure dependent transport parameters.

## 2. Materials and Methods

### 2.1. Materials

Styrene-butadiene-styrene triblock copolymer (SBS, 30 wt % styrene, MW 140.00 g mol^−1^) was purchased from Sigma-Aldrich. Toluene (reagent grade), used for dissolving the SBS without further purification, was purchased from Carlo Erba. The porous support poly (vinylidene fluoride) (PV350) 75 kDa was supplied by Nanostone Water. The Polymer of Intrinsic Microporosity, PIM-SBF-1, was synthesised as described previously [36]. Pure gases (>99.99% purity) and a certified mixture (N_2_/CO_2_/O_2_ with 79.88 mol %, N_2_ 10.10 mol %, CO_2_ and 10.02 mol % O_2_) were supplied by SAPIO (Italy).

### 2.2. Membrane Preparation

#### 2.2.1. Dense SBS Film Preparation

SBS dense membranes were prepared according to the procedure described previously [38,39], dissolving 20 wt % of the polymer in toluene under magnetic stirring at room temperature, generally 23 ± 2 °C. After 24 h, the solution was poured inside a stainless steel casting ring on a glass plate. A dense self-standing membrane was formed after complete solvent evaporation at room temperature for 24 h.

#### 2.2.2. SBS Thin Film Composite Membrane Preparation

Thin film composite membranes were prepared by an analogous procedure, such as that reported by Bazzarelli et al. [38]. The SBS solution in toluene was coated onto the PVDF support by an Elcometer film applicator with a casting gap of 100 µm at room temperature, followed by solvent evaporation for at least 24 h under atmospheric conditions.

#### 2.2.3. Dense PIM-SBF-1 Film Preparation

PIM-spirobifluorene was synthesised as described previously [36]. The polymer was dissolved in chloroform and was cast into a glass petri-dish and left to allow complete solvent evaporation in at least 3–5 days.

### 2.3. Pure Gas Permeation Measurements

Permeation tests with six pure gases were performed as a reference by a fixed volume-pressure increase instrument (ESSR, Geesthacht, Germany) to define the materials performance under standard conditions. The experiments were performed at 25.0 ± 0.5 °C and at 1 bar. The gases were always tested in the same order (He, H_2_, N_2_, O_2_, CH_4_ and CO_2_), although repeated measurements usually showed that the gas order was irrelevant at such low pressures. Before the first test, the membrane was evacuated for at least 1 h, or more if necessary, to guarantee the complete removal of all gases dissolved in the membrane. The correct starting condition of the membrane was checked and confirmed by the absence of significant baseline drift. To guarantee a complete evacuation of the membrane during the test cycle, it was kept under high vacuum for at least ten times the time lag of the previous gas tested. The permeabilities, *P*, are reported in Barrer (1 Barrer = 10^−10^ cm^3^_STP_ cm cm^−2^ s^−1^ cm Hg^−1^) and the values are calculated from the steady state of the permeation curve. The detailed measurement procedure, experimental setup, and data elaboration procedure were reported recently [35].

### 2.4. Mixed Gas Permeation Measurements

Mixed gas permeation experiments were performed by a custom-made constant pressure/variable volume instrument. The samples were placed in a modified Millipore permeation cell and the composition of the permeate was analysed with a Mass Spectrometric (MS) device equipped with a quadrupole mass filter (HPR-20 QIC Benchtop residual gas analysis system for max. 200 AMU, Hiden Analytical, Warrington, UK) and a sampling capillary with a typical flow rate of ca. 12 cm^3^ min^−1^ argon at ambient pressure. The feed pressure (0–5 bar(g)) was controlled by an EL-PRESS electronic back pressure controller (Bronkhorst High-Tech, Ruurlo, The Netherlands) and the feed (200–500 cm^3^ min^−1^) and the sweep (typically 30 cm^3^ min^−1^) flow rates were controlled by EL-FLOW electronic Mass Flow Controllers (Bronkhorst High-Tech, Ruurlo, The Netherlands). The measured mass spectrometer data were recorded with the MASsoft 7 software (Hiden Analytical, Warrington, UK) package supplied with the mass spectrometer, while the pressure and flow rates were recorded with the FlowPlot software (Bronkhorst High-Tech, Ruurlo, The Netherlands) supplied with the pressure and mass flow controllers. Data elaboration was carried out by a custom-written macro in MS Excel. The development, error analysis and validation of this method with a detailed description of the procedures were reported previously [35].

## 3. Results and Discussion

### 3.1. System Response and Setup

**Figure 2:**
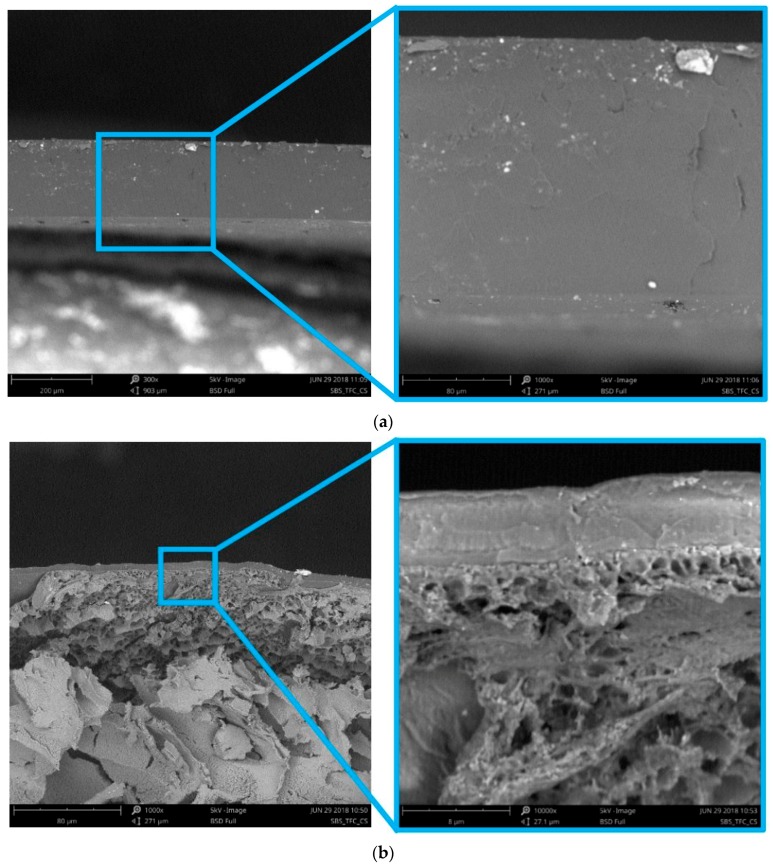
SEM image of the 159 µm thick dense SBS membrane (**a**); the 5 µm thin film composite SBS membrane (**b**).

A fundamental aspect of the on-line analysis of the permeate composition is the sufficiently fast response time of the system. In our previous work, the instrument response time was studied for Pebax^®^2533 and Hyflon^®^AD60X membrane samples, as well as an aluminium film with a pinhole, and resulted to be approximately 20 s [35]. In this work, further evaluation and optimisation of the setup is carried out with a thin film composite (TFC) membrane with an effective thickness of 5 µm of the rubbery block copolymer SBS and a thick dense film (159 µm) for comparison (Figure 2). The active layer of the TFC membrane is thin enough to have a negligible time lag, and therefore it is used to verify the integrity of the system and the membrane, through measurement of the selectivity, and to determine the instrumental time lag, through measurement of the permeation transient and steady state. Both the TFC membrane and the thick SBS film appear completely dense by the SEM analysis (Figure 2), without visible defects, as later confirmed by their permselectivity.

#### 3.1.1. Instrumental Time Lag at Atmospheric Feed Pressure

The effect of the sweep flow rate on the thick dense SBS film and the thin film composite membrane is shown in SI Figure S1. As foreseen by Equation (8), the time lag increases with decreasing sweep flow rate. All slopes are equal within the range of the experimental error, with a slightly lower slope for CO_2_ in the TFC as the only exception. The reason for the lower slope of CO_2_ is not fully understood, but since the permeance (SI Figure S1b) and the permselectivity (SI Figure S1c) are nearly constant, and since the permeance of the most permeable gas, CO_2_, does not decrease with decreasing sweep flow rate, it may be assumed that there are no significant polarization phenomena at the downstream side of the membrane. This confirms SBS to be a suitable material for further optimization studies. The thin film composite SBS membrane has a negligible time lag, and the observed values correspond to the instrumental time lag, whereas the difference with the thicker membrane accounts for the time lag of the SBS membrane itself. The slope in the curves of SI Figure S1a allows for the determination of *V_Downstream_*, which is one of the volumes that contribute to the instrumental time lag.

#### 3.1.2. Instrumental Time Lag at Variable Feed Pressure

**Figure 3:**
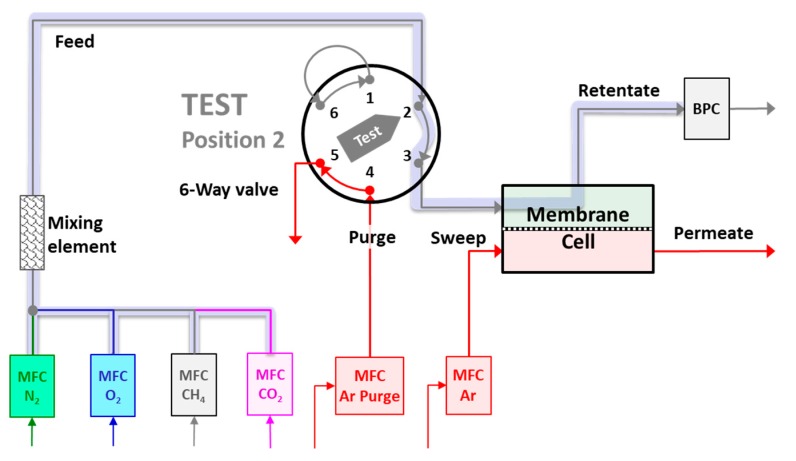
Details of the experimental setup, highlighting with the thick shaded grey line the feed section, which determines the pressure increase rate (adapted from Fraga and Jansen et al. [35]).

A limitation of the previous work is that the instrument was set up for the analysis of mixed gas permeation transient measurements at atmospheric feed pressure [35]. However, since real separations are always performed at higher pressures to provide the desired driving force, knowledge of the response of the system under such conditions is very important. This response is fundamentally different, as can be seen from the schematic image of the feed section of the instrument in Figure 3. The response time for switching from argon to the feed gas at atmospheric conditions is dictated only by the ratio of the feed flow rate and the very small volume between the six-way valve at point 3 and the membrane cell. In contrast, the response at higher pressures depends on the entire pipe volume from the mass flow controllers (MFCs) until the back pressure controller (BPC).

**Figure 4:**
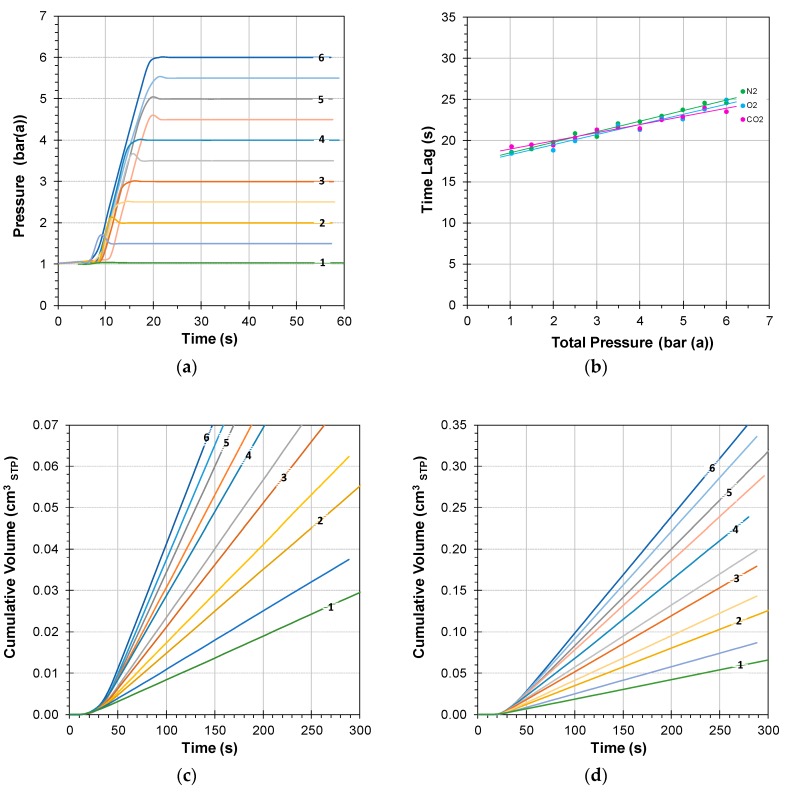
Plot of the increase of the feed pressure as a function of time upon switching of the six-way feed valve in Figure 3 from Argon purge gas to the 80/10/10 vol % N_2_/CO_2_/O_2_ mixture, mimicking CO_2_-poor flue gas (**a**), and corresponding instrumental time lag (**b**) determined with an SBS TFC membrane (area 1.77 cm^−2^). Examples of the individual time lag curves for N_2_ (**c**) and CO_2_ (**d**). Feed flow rate 500 cm^3^_STP_ min^−1^ and sweep flow rate 30 cm^3^_STP_ min^−1^. The numbers on the curves in (**a**,**c**) and (**d**) indicate the feed pressure in bar(a); the intermediate curves are the half-integer values.

The setpoint of the pressure is set to the desired value exactly when the six-way valve switches from argon purge to the feed gas, and the response of the system at different feed pressures is reported in Figure 4. After an initial delay for the BPC to react, there is a constant pressure increase rate, defined by:

(9) $$\frac{dp_{T}}{dt}=\frac{\phi_{\mathrm{Feed},T}}{V_{\mathrm{Total},\mathrm{Feed}}}$$

and at a given volumetric flow rate, the unknown volume can be calculated as:

(10) $$V_{\mathrm{Total},\mathrm{Feed}}=\frac{\phi_{\mathrm{Feed},T}}{dp_{T}/dt}$$

In the present setup, the total time needed to reach the maximum pressure of 6 bar(a) is approximately 12 s, starting from 1 bar and at a flow rate of 500 cm^3^ min^−1^. This means that the total dead volume in the system that must be pressurised is ca. 20 cm^3^. Inversely, the time needed for a certain pressure increase, Δ*p*, is given by:

(11) $$t=\frac{V_{\mathrm{Total},\mathrm{Feed}}}{\phi_{\mathrm{Feed},T}}\Delta p$$

**Figure 5:**
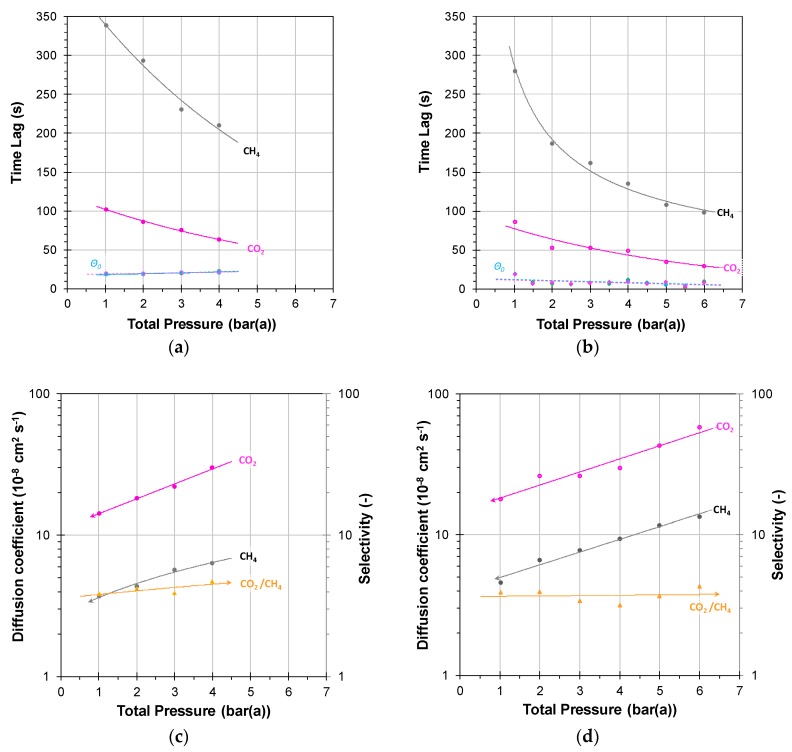

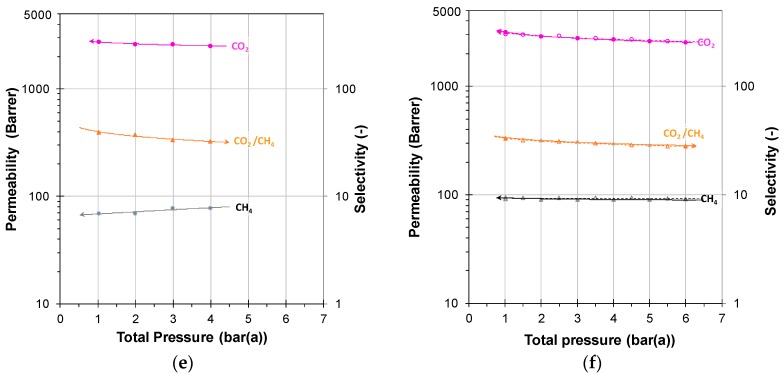
Individual time lag for CO_2_ and CH_4_ in a 2088 days aged sample of PIM-SBF-1 with individual pressure steps (**a**) and a stepwise incremental pressure increase (**b**). The instrumental time lag at the bottom of the graphs (**a**,**b**) needs to be subtracted for the calculation of the corresponding diffusion coefficients (**c**,**d**), showing strong pressure dependence. The permeability and selectivity show a much lower pressure dependence (**e**,**f**). Gas mixture 35/65 vol % CO_2_/CH_4_.

Unlike the average residence time in the tubes and other volumes in the setup that contributes for 100% to the instrumental time lag (Equation (7)), the time needed for the pressure increase contributes less to the overall time lag because permeation already starts before the maximum pressure is reached. Indeed, Figure 4b shows that for higher feed pressures, the time lag increases only 6–7 s. This is a small, but significant, contribution, considering that the standard deviation in the time lag measurements is in the order of 2 s [35]. The apparent difference in the trend of O_2_, N_2_, and CO_2_ is most likely due to the different flow regime in the narrow sampling capillary and in the low pressure quadrupole inlet, where Knudsen diffusion plays a role. This difference is negligible compared to the membrane-related time lag (Figure 5a,b), and since it falls more or less within the range of the experimental error, it does not affect any of the conclusions.

At a lower feed flow rate of 200 cm^3^_STP_ min^−1^, the filling-up time is ca. 25 s at 5 bar (SI Figure S2a), however, over this pressure interval the instrumental time lag increases only 10–12 s (SI Figure S2b). Thus, for the total time lag:

(12) $$\Theta_{i}=\Theta_{0}+\Theta_{Mem,i}=\frac{V_{Feed}}{\phi_{Feed}}+\frac{V_{Downstream}}{\phi_{Downstream}}+\frac{V_{Inlet}}{\phi_{Inlet}}+\Theta_{\Delta p}+\frac{l^{2}}{6D_{i}}$$

where *Θ*_Δ*p*_ is the additional time lag induced by the slow pressure increase, and:

(13) $$\Theta_{\Delta p}<\frac{V_{\mathrm{Total},\mathrm{Feed}}}{\phi_{\mathrm{Feed},T}}\Delta p$$

At low feed flow rates, a longer time is needed to reach the desired pressure (SI Figure S2a), resulting in a slower overall response (SI Figure S2b) and a higher instrumental time lag (SI Figure S2c). In further experiments, the maximum possible feed flow rate is therefore used until the desired pressure is reached, and then the feed flow rate is reduced to 200 cm^3^_STP_ min^−1^.

### 3.2. Mixed Gas Diffusion in the Polymer of Intrinsic Microporosity PIM-SBF-1

#### 3.2.1. Individual Pressure Steps

The above procedure was applied to measure the permeation transient and steady state of CO_2_/CH_4_ mixtures in PIM-SBF-1 at different pressures, and the results are given in Figure 5. All experiments were performed on a well-aged sample (2088 days) to avoid time-dependent phenomena. At high feed flow rates of 500 cm^3^ min^−1^, the feed pressure during permeation experiments at 1–4 bar still requires 10–15 s to reach the setpoint value (SI Figure S4a). This time is not negligible and contributes, to some extent, to the overall time lag. Figure 5a shows the measured overall individual time lags for CO_2_ and CH_4_, and the instrumental time lag, measured with the TFC membrane of SBS, is plotted for comparison as the lower blue line in Figure 5a. For a similar problem in gravimetric sorption measurements, where significant time is needed to fill the sorption chamber, Vopička et al. proposed a mathematical correction of the model [40]. In the present work, we assume additivity of each characteristic time in the procedure. Therefore, we subtract the time lag for the TFC membranes from that of CO_2_ and CH_4_ at the same pressure. Rearranging of Equation (12) then allows for the calculation of the diffusion coefficient (Figure 5c) of each gas according to:

(14) $$D_{i}=\frac{l^{2}}{6\cdot (\Theta_{Mem,i}-\Theta_{0})}$$

It must be noted that the permeate flow rate (e.g., SI Figure S3) must be low enough compared to the sweeping gas flow rate to have negligible impact on the calculation of the time lag in all experiments. Taveira et al. [27] calculated that for p_F,i_/p_P,I_ > 40–50 the error in *D* is less than 5%. In the present work, this ratio was generally higher so that the inaccuracy in the determination of *D* due to the changing boundary conditions in the downstream side is even lower than 5%.

In contrast, the gas permeability, determined from the steady-state permeation after each feed pressure step, is only slightly dependent on the feed pressure (Figure 5e), and the diffusion coefficients of both CO_2_ and CH_4_ show a very distinct and nearly exponential trend with increasing feed pressure. Both increase two-fold in the range from 1 to 4 bar(a), with only a marginal increase in the diffusion selectivity. In the same pressure interval, the CO_2_/CH_4_ permselectivity decreased about 20% from ca. 40 to 32 as a result of a 10% decrease of the CO_2_ permeability and a 10% increase of the CH_4_ permeability. An increase in diffusivity at nearly constant permeability means that the solubility, *S*, decreases with increasing pressure, considering that permeability is the product of solubility and diffusivity:

(15) $$P_{i}=D_{i}\cdot S_{i}$$

This agrees well with the dual mode sorption (DMS) mechanism or the Guggenheim, Anderson, and de Boer (GAB) layered-adsorption model that typically describes the sorption behaviour of PIMs [41]. In these models, the solubility gradually decreases with increasing pressure as more and more of the available sorption sites are occupied. Of course, as discussed in the introduction, the time lag method strictly gives only correct results for membranes with linear Henry type sorption behaviour and concentration-independent diffusion. Thus, the strong nonlinearities observed clearly mean that the calculated permeability and diffusion coefficients are effective values, averaged over the thickness of the membrane and valid for the given experimental conditions. Precise analysis of pressure- and concentration-dependent transport parameters would require much more complicated and laborious experimental and computational methods. Nevertheless, the strength of the time lag method, which explains its extreme popularity, is its simplicity from the experimental point of view and the capacity to pinpoint qualitative trends when comparing different materials or different conditions.

#### 3.2.2. Incremental Pressure Steps

As a further demonstration of the strength of the method, the possibility to apply incremental pressure steps rather than the more time consuming single pressure steps was investigated. As an example of this procedure, SI Figure S3 shows the feed pressure, the flow profiles, and the response of the permeate flow rate for a representative experiment with the SBS TFC membrane. For PIMs with fast permeation kinetics, this step-wise procedure has the clear advantage that it requires only 5 s to reach the setpoint for each pressure step (SI Figure S4b), making the dynamics of the incremental procedure significantly faster. Similar to the procedure with the individual steps, it is possible to calculate a time lag in each subsequent pressure increase step. The results in Figure 5b show that the time lag measured by this procedure is somewhat shorter than that with the individual steps for CH_4_, but nearly identical for CO_2_ (Figure 5a). This gives an excellent qualitative impression of the pressure dependence of the diffusion coefficients and the diffusion selectivity (Figure 5c,d). However, since the boundary conditions for which Equations (2)–(5) are valid, namely, that the membrane is penetrant-free at the beginning of the experiment, are not satisfied in the second and subsequent pressure steps, quantitative interpretation of these data must be done with care. This procedure would require a correction for the initial concentration of the penetrant [42] that takes into account the finite non-zero initial gas concentration in the membrane before each pressure step. After the first step, there is already a concentration gradient across the membrane, corresponding to the steady state permeation at the previous pressure, as schematically displayed in SI Figure S5. Theoretically, also the desorption kinetics would allow the calculation of the diffusion coefficient. Simple application of Equations (2)–(5), without appropriate corrections for the boundary conditions, gives the same trend with shorter time lags and, thus, apparently faster diffusion than for the pressure increase run (SI Figure S6).

The permeability and selectivity are measured in steady state for each pressure step and besides minor differences due to a change of the properties as a function of time by aging or pressure induced dilation, these values are independent of transient phenomena and are essentially identical for both methods (Figure 5e,f). In the case of pressure-dependent permeation, the concentration gradient across the membrane is not linear. In this situation, the time lag method has the practical limitation that it only gives an effective permeability and diffusion coefficient, averaged over the entire membrane. The solution of this problem requires complex numerical methods, but for qualitative analysis of the effect of pressure, temperature, composition, or other variables on the transport properties, the time lag approach remains a simple and powerful method.

## 4. Conclusions

On-line measurement of transient and steady state permeation of gas mixtures by mass spectrometry allows the determination of the diffusion coefficient of individual components in gas mixtures. An appropriate correction is needed for the instrumental time lag related to the average residence time of the gases in the tubing and various sections of the instrument. Measurements at elevated pressure require an additional correction for the time needed to reach the setpoint of the feed gas pressure. This correction is shorter than the extra residence time of the gas in the system because permeation already starts as soon as the pressure begins rising. Such gradual exposure of the membrane to increasingly higher pressures would require a complex mathematical adjustment of the time lag model with variable boundary conditions. However, a satisfactory solution is the experimental analysis of the instrumental time lag with a thin film composite membrane under the same conditions, followed by subtraction of this value from the experimental time lag of a thick dense membrane.

With an uncertainty of a few seconds in the time lag, this method can provide highly accurate diffusion coefficients of any common gas in dense membranes. In the example of the polymer of intrinsic microporosity, PIM-SBF-1, CO_2_ demonstrated to have an approximately five times higher diffusion coefficient than CH_4_, which both increase by a factor of two when increasing the feed pressure from 1 to 4 bar(a), with a marginal increase in the diffusion selectivity. Instead, in the same pressure interval, the CO_2_/CH_4_ permselectivity decreased about 20% from 40 to 32, as a result of a 10% decrease of the CO_2_ permeability and a 10% increase of the CH_4_ permeability. This means that the solubility must show the opposite effect and thus decrease with increasing pressure, as generally observed for PIMs with a strong dual mode sorption behaviour.

Preliminary measurements with a step-wise increase of the feed pressure allow a similar analysis of the transient phenomena for each pressure step and suggest that this may be used as a quicker and more versatile method to determine the pressure-dependent permeation transient, and thus the mixed gas diffusion coefficients. The different boundary conditions, where the membrane is not free of any gas before the measurement, requires a different mathematical treatment of the data, which will be the subject of further research.

## Supplementary Materials

The following are available online at http://www.mdpi.com/2077-0375/8/3/73/s1, SI Figure S1. Effect of the sweep flow rate on the time lag (A), the permeances (B) and the permselectivity (C) of a fast 5 µm thin film composite membrane (spheres) and a slower 159 µm thick dense film (diamonds) for the N2/O2/CO2 80/10/10 vol% mixture. Sweeping gas at atmospheric pressure; SI Figure S2. Increase of the feed pressure upon switching of the 6-way feed valve from Argon purge to the 80/10/10 vol% N2/CO2/O2 mixture (a), corresponding permeation curves for O2 (b) and resulting instrumental time lag (c) determined with an SBS TFC membrane (area 1.77 cm^−2^). Feed flow rate 200 cm3STP min^−1^ and sweep flow rate 30 cm3STP min^−1^; SI Figure S3. Profile of the feed flow rates and feed pressure (A), and the permeate flow rates (B) as a function of time for the SBS thin film composite membrane with a 80/10/10 vol% N2/CO2/O2 mixture; SI Figure S4. Comparison of the increasing feed pressure as a function of time for individual pressure increase ramps (A) and for stepwise increasing pressure (B) for 2088 days aged sample SBF with the gas mixture CO2/CH4 35/65 vol%; SI Figure S5. Schematic representation of the development of the concentration profiles in the membrane after its first exposure to the gas and during two subsequent pressure increase steps; SI Figure S6. Individual time lag for CO_2_ and CH_4_ in a 2088 days aged sample of PIM-SBF with a stepwise pressure increase (full circles). The empty circles show the corresponding points for the pressure decrease steps. Points of the instrumental time lag Θ_0_ for N_2_, O_2_ and CO_2_ overlap.
