# Supplementary material for: A Novel Time Lag Method for the Analysis of Mixed Gas Diffusion in Polymeric Membranes by On-Line Mass Spectrometry: Pressure Dependence of Transport Parameters
Source: Membranes (Basel). 2018 Sep 3;8(3):73. doi: 10.3390/membranes8030073 (PMC6161161; doi:10.3390/membranes8030073)
Supplement: Supplementary file 1 [file membranes-08-00073-s001.pdf]

membranes

Supplementary Materials

1. Analysis of the Instrumental Time Lag for a Thick Dense SBS Film and an Sbs TFC Membrane

SI Figure 1 gives a comparison of the time lag both , permeance, and permselectivity of a thick SBS film and a TFC SBS membrane. The trends for membranes are very similar and the small difference in the selectivity is probably due to the slightly different properties in bulk and in thin films. The virtually constant permeability and selectivity as a function of the sweep flow rate suggest that polarization phenomena in the permeate side are negligible, as this would have led to a decrease in permeability of the most permeable gas (CO_2) at the lowest sweep flow rates.

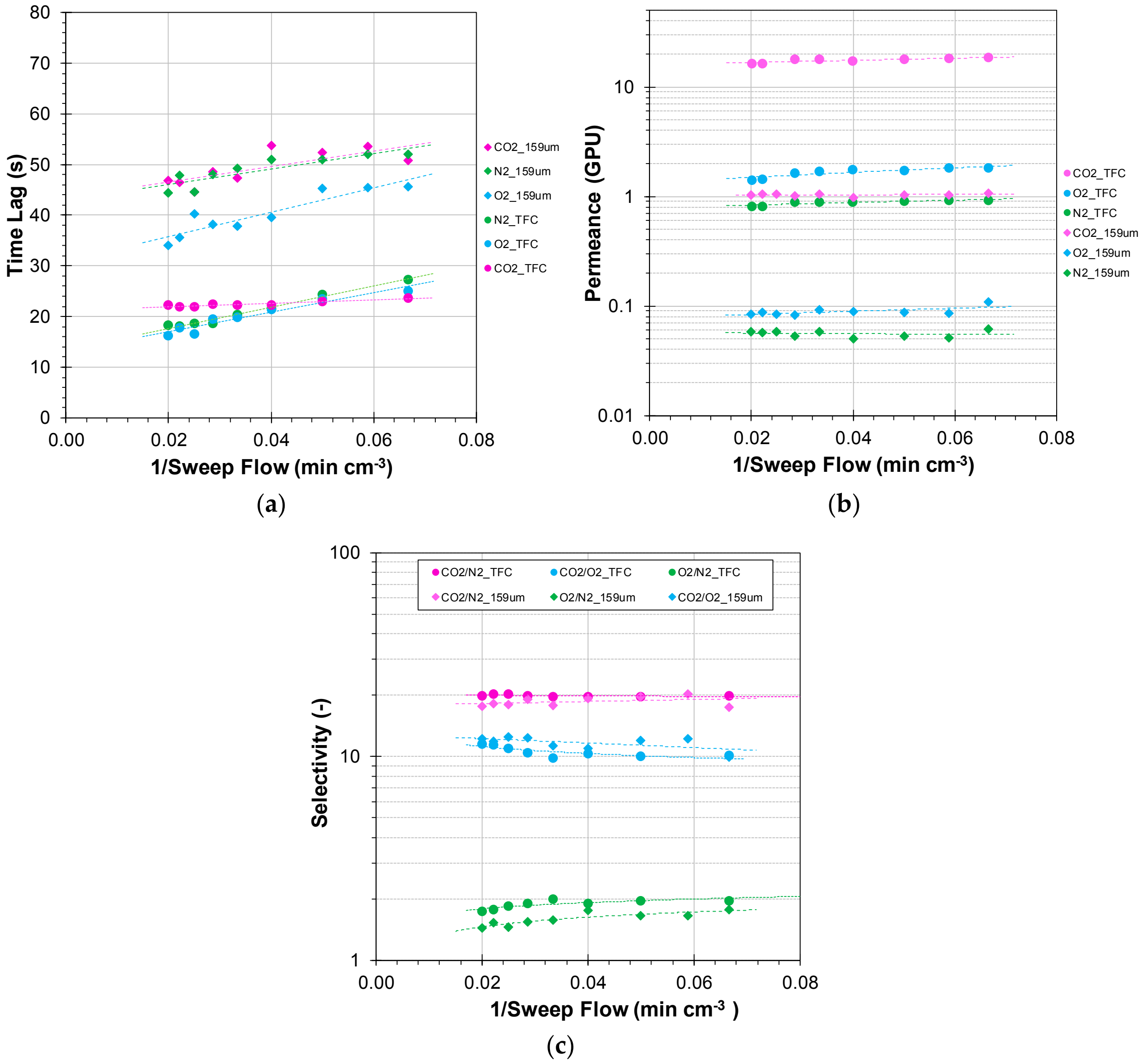

SI Figure 1. Effect of the sweep flow rate on the time lag (**a**), the permeances (**b**), and the permselectivity (**c**) of a fast 5 µm thin film composite membrane (spheres) and a slower 159 µm thick dense film (diamonds) for the $N_2/O_2/CO_2$ 80/10/10 vol % mixture. Sweeping gas at atmospheric pressure.

2. Study of the Effect of Slow Pressure Increase on the Instrumental Time Lag

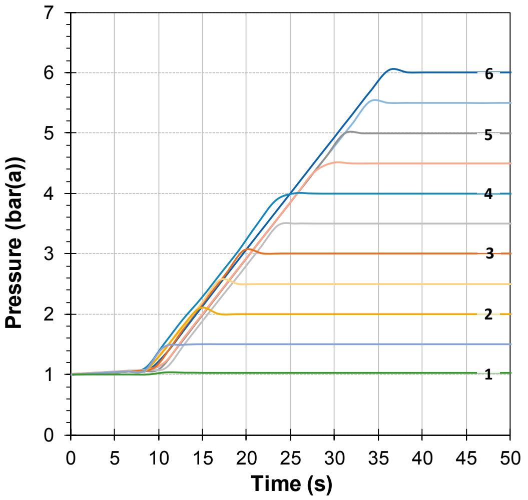

(a)

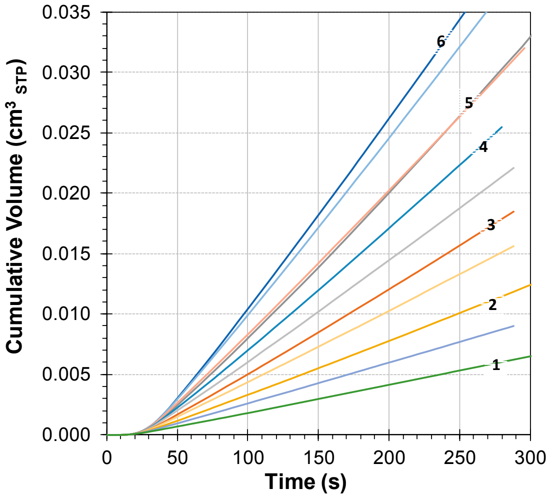

(b)

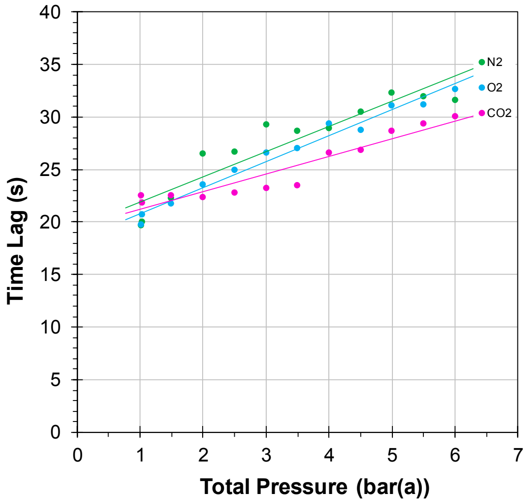

(c)

SI Figure 2. Increase of the feed pressure upon switching of the six-way feed valve from Argon purge to the 80/10/10 vol % $N_2/CO_2/O_2$ mixture (**a**), corresponding permeation curves for O_2 (**b**), and resulting instrumental time lag (**c**) determined with an SBS TFC membrane (area 1.77 cm^{-2}). Feed flow rate 200 cm^3_{STP} min^{-1} and sweep flow rate 30 cm^3_{STP} min^{-1}.

3. Reaction of the Permeate Composition on Changes in the Feed Pressure

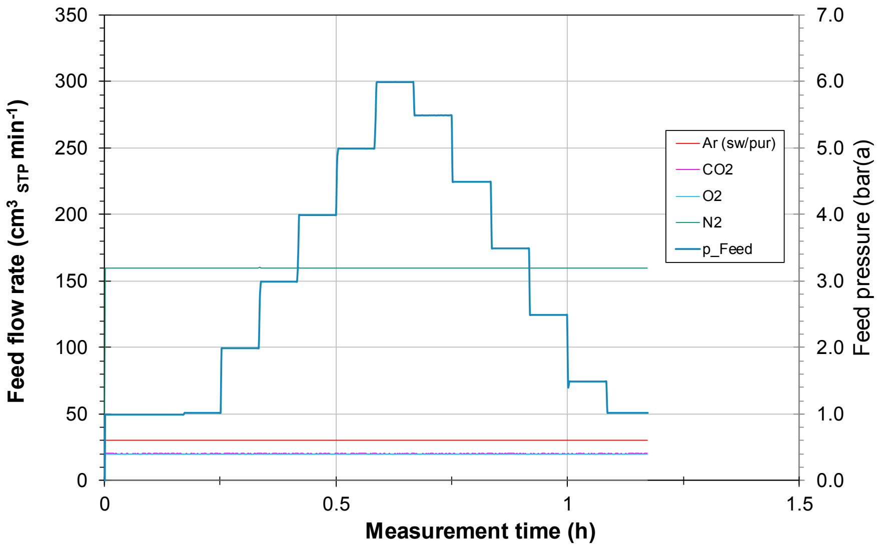

(**a**)

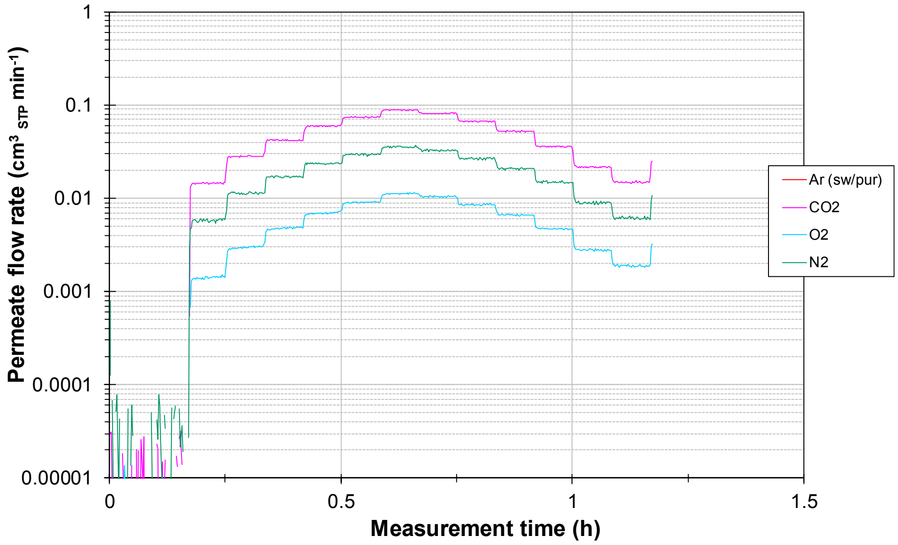

(**b**)

SI Fig. 3. Profile of the feed flow rates and feed pressure (**a**), and the permeate flow rates (**b**) as a function of time for the SBS thin film composite membrane with a 80/10/10 vol % $N_2/CO_2/O_2$ mixture.

4. Mixed Gas Diffusion in the Polymer of Intrinsic Microporosity PIM-SBF-1

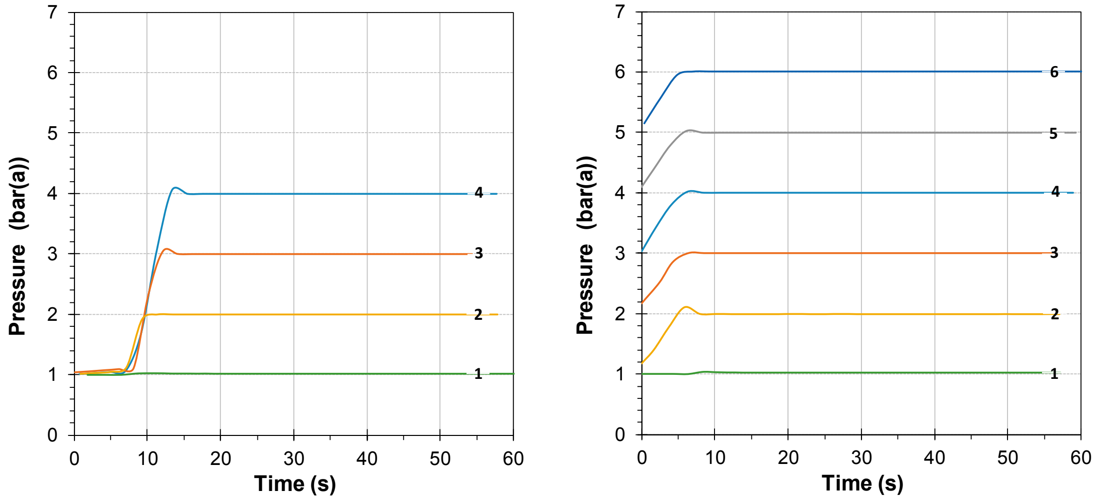

(**a**) (**b**)

SI Figure 4. Comparison of the increasing feed pressure as a function of time for individual pressure increase ramps (**a**) and for stepwise increasing pressure (**b**) for 2088 days aged sample SBF with the gas mixture CO_2/CH_4 35/65 vol %.

5. Schematic Concentration Profile Development in Three Subsequent Pressure Steps

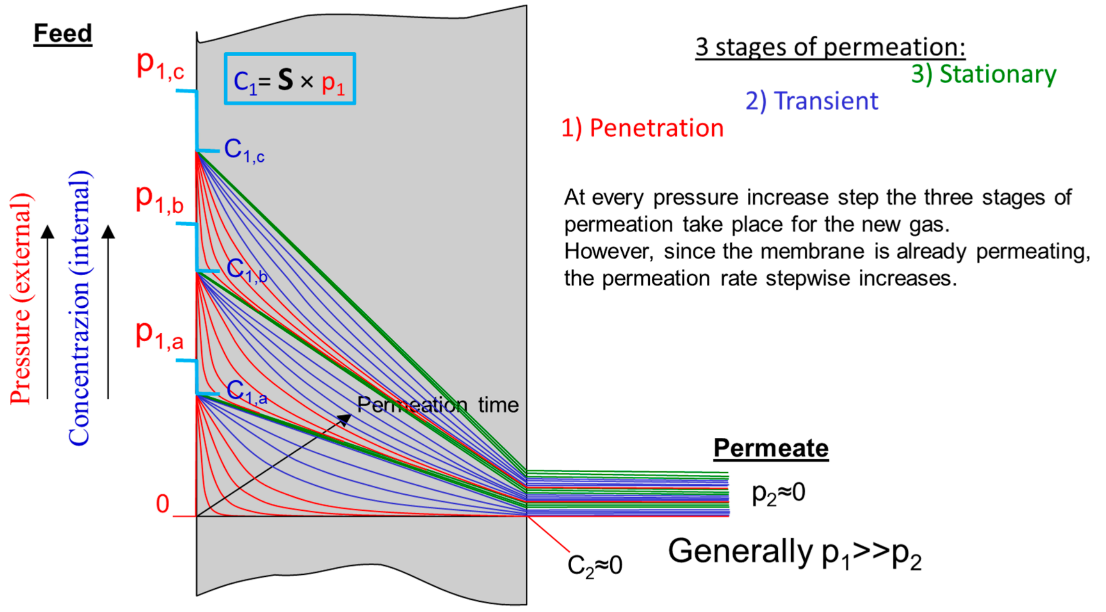

SI Figure 5. Schematic representation of the development of the concentration profiles in the membrane after its first exposure to the gas and during two subsequent pressure increase steps.

6. Apparent Time Lag during Pressure Increase Steps and Pressure Decrease Steps

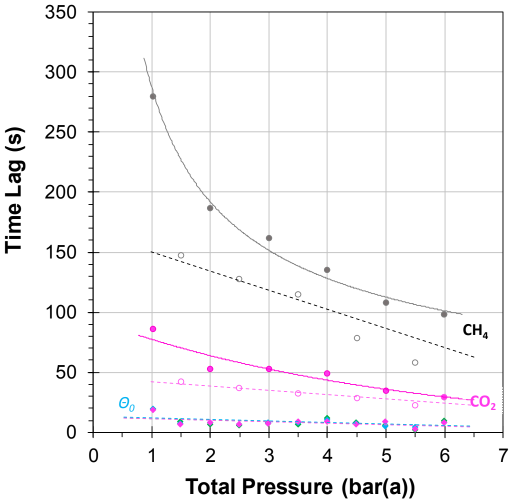

SI Figure 6. Individual time lag for CO_2 and CH_4 in a 2088 days aged sample of PIM-SBF with a stepwise pressure increase (full circles). The empty circles show the corresponding points for the pressure decrease steps. Points of the instrumental time lag Θ_0 for N_2, O_2, and CO_2 overlap.
